# A Roadmap for Implementing Virtual Geriatric Mental Health Services for Rural Veterans: Qualitative Longitudinal Evaluation

**DOI:** 10.2196/95286

**Published:** 2026-06-16

**Authors:** Christine E Gould, Marika Blair Humber, Chalise Carlson, Ranak B Trivedi, Marisa-Francesca Lindsey, Althea Lloyd, Lynsay Paiko, Amanda D Peeples

**Affiliations:** 1 Geriatric Research Education and Clinical Center, VA Palo Alto Health Care System, 3801 Miranda Ave, Palo Alto, CA, 94304, United States, 1 650-4935000; 2 Veterans Rural Health Resource Center - Salt Lake City (VRHRC-SLC), VA Salt Lake Health Care System, Salt Lake City, UT, United States; 3 Department of Psychiatry & Behavioral Sciences, Stanford University School of Medicine, Stanford, CA, United States; 4 Center for Innovation to Implementation, VA Palo Alto Health Care System, Menlo Park, CA, United States; 5 Clinical Resource Hub, Veterans Integrated Service Network (VISN) 7, Atlanta, GA, United States; 6 Center for Healthcare Engagement, Research, and Promotion (CHERP), Corporal Michael J. Crescenz VA Medical Center, Philadelphia, PA, United States

**Keywords:** CFIR, rural health, older adults, telehealth, virtual care

## Abstract

**Background:**

Access to geriatric mental health (GMH) care is limited in rural areas. To meet this need, the Veterans Health Administration provides specialty tele-GMH care for aging rural veterans via regional telehealth hubs.

**Objective:**

This study aims to create a roadmap describing key phases and determinants underlying the implementation and sustainment of tele-GMH services as part of a qualitative longitudinal evaluation of tele-GMH teams.

**Methods:**

Semistructured interviews were conducted with clinicians from all 8 tele-GMH teams (n=25) at 3 time points across a 3-year period (October 2021-September 2024). Interview (n=46) data were summarized into key domains using a templated rapid qualitative approach, guided by the Consolidated Framework for Implementation Research (CFIR) 2.0. Further thematic analysis and team discussion elucidated the findings.

**Results:**

We identified key activities and determinants of success in three phases: (1) preimplementation (engaging leaders, securing funding/hiring, and defining services); (2) implementation scale-up and expansion (advertising, addressing challenges, seeking feedback, refining, and growth); and (3) sustainment (maintenance). Activities within each phase were cyclical and iterative (ie, nonlinear). Barriers to implementation included unfamiliarity with local aging resources; facilitators included tailoring strategies and engaging referring clinicians.

**Conclusions:**

Similar processes emerged across regions in the development and sustainment of tele-GMH services, allowing for the creation of a unified roadmap. Limitations including sampling bias are discussed. Further work could apply and evaluate the utility of the roadmap to guide creation of tele-GMH services in new regions to enhance access to specialty care for aging rural veterans.

## Introduction

Over half (54%) of US veterans residing in rural areas are aged 65 years or older and face challenges in accessing and coordinating health care services [1]. These problems are compounded for aging rural veterans who experience complex mental health, neurocognitive, and medical needs that warrant specialized geriatric mental health (GMH) services. Access to GMH specialists is scarce, particularly in rural areas [2,3], but virtual care (ie, video/audio telehealth services and e-consultation) overcomes geographical barriers to accessing these services [4-6]. Virtual care has been demonstrated to be acceptable, feasible, and efficacious for older patients, caregivers, and referring clinicians [7-9]. However, barriers such as access to broadband internet and digital literacy attenuate the positive impact of virtual care for some rural veterans [10].

The Veterans Health Administration (VHA), the largest integrated health care system in the United States, is a leader in the provision of telehealth services [11] and in addressing barriers to care through national programs, such as a Digital Divide Consult through which clinicians can request that veterans receive loaned video-enabled tablets with internet access [12]. The VHA also created regional telehealth centers to deliver virtual care to multiple health care systems in a VHA region. These regional telehealth centers, expanded broadly under the nomenclature of clinical resource hubs (CRHs), address gaps in VHA primary and mental health care access due to clinical staffing shortages caused by resignations, extended leave periods, difficulty hiring needed clinicians, or increasing enrollment of veterans [13,14]. CRH services, which initially focused on temporary clinical support based on requests from local VHA medical centers or clinics, have expanded to encompass specialty care such as cardiology [15], sleep medicine [16], and specialty mental health models (eg, tele-neuropsychology) that provide consultative care [17]. Other VHA programs that provide virtual care to older veterans occur as part of regular care from VHA medical centers, outpatient clinics, and geriatrics centers of excellence to rural clinics [18,19]. However, CRH-based services differ from other services in VHA in that these regional hubs have clinicians focused only on delivering virtual care and embedded support staff specifically trained to facilitate telehealth services [13].

One CRH specialty model is tele-GMH. Tele-GMH refers to the provision of pharmacological and nonpharmacological management of neurocognitive and psychiatric conditions in older adults with multiple medical conditions by one or more GMH clinicians via virtual modalities. One such tele-GMH consultation model led by a geriatric psychiatrist in a multistate rural region was the pioneering innovation that led to this evaluation [20,21]. This model of care mitigated perceived barriers impeding access to specialty geriatric psychiatry care, including geographic barriers and limited workforce [2,3], and was valued by caregivers and referring clinicians [22]. Regional needs and leadership preferences contributed to the initial development of diverse approaches to tele-GMH care, including single clinician-led services and interdisciplinary teams and models of care [23].

Best practices [24,25] and drivers of telehealth use, such as patient and clinician factors [26,27], have been identified; however, systematic examination of factors supporting implementation and growth of tele-GMH services is needed. We conducted a naturalistic prospective program evaluation (ie, systematic collection of data on the program to assess impact, acceptability, and implementation determinants) using qualitative methods guided by the Consolidated Framework for Implementation Research (CFIR) 2.0 [28]. CFIR allows for consideration of the complex setting within which care is delivered, spanning local clinics, virtual telemedicine hubs, and the larger VHA system. The objective of the evaluation was to identify barriers and facilitators of implementation and to synthesize findings across multiple sites to create an implementation roadmap to facilitate the future spread of tele-GMH services to additional VHA regions.

## Methods

### Study Design

This program evaluation used a qualitative longitudinal design, collecting data across different stages of development and implementation of the tele-GMH teams over a 3-year period. Interviews were conducted between February 2022 and September 2024 via convenience sampling. The initial study design was guided by CFIR 1.0 [29]; however, the study design was updated, and all analysis was conducted based on CFIR 2.0 [28]. Findings reported are based on CFIR 2.0.

### Setting/Participants

We used a convenience sampling approach, focused on trying to reach all the clinicians from the 8 CRHs that provided GMH services or specialty geriatrics services between October 2021 and September 2024 to participate in one or more interviews. We invited 26 of the 27 clinicians; we were unable to invite one clinician because they left their VHA position before the invitation was sent. All clinicians served rural veterans, as identified using the rural-urban commuting area codes used by the VHA Office of Rural Health [1]. We refer to the services as tele-GMH, as the majority of clinicians and services are focused on GMH.

### Measures

Led by the project director (CEG), the analysis team (MBH, ADP, and RBT) developed semistructured interview guides to be administered to clinicians at 3 time points corresponding to each service’s startup (time point 1), development (time point 2), and sustainment (time point 3) stages. Interview guides are available from the first author (CEG) by request; the overarching purpose of these guides was related to the multiyear evaluation with varying data collected at each time point.

During the time point 1 interviews, clinicians were asked about factors related to the development of their service, reasons why they joined the CRH, services provided, common reasons for referral and referral sources, regularly used measures, team composition, and team collaboration. Overall, time point 1 focused on CFIR domains related to the innovation and characteristics of the inner setting.

Time point 2 interviews asked clinicians about planning and implementation of services, facilitators and barriers of implementation, service impacts on rural veterans and their caregivers, service impacts on referring clinicians and others in the health care system, outcomes to track, and reflections on the sustainment of the service. This interview guide included questions that mapped onto constructs in CFIR 1.0. Time point 2 interviews gathered information about changes to the innovation and the inner setting and the initial implementation process.

Time point 3 interviews focused on relationships with referral sources, service impacts on referring clinicians and rural veterans, barriers and facilitators to service delivery, and geriatric medicine needs [30] for rural veterans. This interview guide asked questions about any changes to innovation, the inner setting, and the implementation process.

We aimed to interview all clinicians at time point 1, then used more purposive sampling at time points 2 and 3 to ensure that perspectives were gathered from multiple disciplines and from every CRH team. This purposive approach for time points 2 and 3 allowed for gathering perspectives from every region with services while minimizing time burden on the clinicians delivering services.

### Ethical Considerations

The project was reviewed by the Stanford University IRB, the IRB of record for VA Palo Alto Health Care System, and determined that this work did not meet the definition of human subjects research (review numbers: IRB-65150, IRB-70700, and IRB-73787) and program evaluation was conducted to inform VA operations. Union concurrence was obtained prior to conducting interviews. All participants provided verbal consent prior to their virtual interviews.

### Procedures

In accordance with VHA policy, interview guides were sent to the unions for review and concurrence. Participants were emailed an invitation to engage in interviews approximately annually. One reminder email was sent to participants who did not respond to the first invitation. Interviews were conducted and recorded via videoconferencing technology. All interviewers were trained in qualitative interviewing by the qualitative methodologist (ADP).

### Analysis

The evaluation team consisted of a geropsychologist/project director (CEG), project coordinators (CC and MFL), a qualitative analyst (MBH), a qualitative methodologist (ADP), geropsychologist (AL), a health psychologist (RBT), and a geropsychology postdoctoral fellow (LP). Interviews were conducted by 5 authors (CEG, MBH, CC, MFL, and LP). All evaluation team members participated in the analysis (see Multimedia Appendix 1 for reflexivity statements from the evaluation team). Biases were managed through discussion, weekly qualitative analysis meetings, and multiple authors reviewing narrative summaries.

Interviews were auto-transcribed using Microsoft Teams software. Team members proofread transcriptions against the audio recordings to ensure that transcripts were verbatim. Led by the project director (CEG), the team developed templates to summarize transcripts into domains (key topics of interest) that corresponded to the interview guides. Using rapid qualitative template-based analysis [31], one individual summarized each transcript, and a second individual reviewed the transcript summary; then both met to review the summary and resolve any discrepancies by consensus. Data saturation was determined through review of the transcript summaries and the written domain narratives that synthesized data across each summary. Review occurred in pairs; the full team discussed findings during twice-monthly meetings. Domain narratives with similar content were combined. This analysis focused on 8 domain summaries, including joining CRH; name of service; planning process; advertising; determinants; relationships with referral sources; services/challenges; and outcomes, sustainability, and service impacts. Two authors (CEG and MBH) used a matrix template based on CFIR 2.0 [28,32] to code the domain summaries to identify the key components of CFIR underlying our findings. These 2 authors also used the 8 domain narratives to generate the implementation roadmap. The roadmap was reviewed and refined during qualitative team meetings. The finalized implementation roadmap was reviewed with the project advisory team, which included 2 CRH geriatric psychiatrists, a CRH director, and a national leader in GMH.

The evaluation team developed materials summarizing key aspects related to the development and implementation of tele-GMH services in the CRHs, outlining steps sites may consider in the planning and implementation process. These materials were reviewed with CRH clinicians during monthly consultation meetings and were distributed to CRH clinicians via email. Minor changes (focused primarily on CRH-specific processes) were made based on feedback. This process enabled the evaluation team to engage in member checking [33] through sharing our understanding of implementation processes with the interviewees themselves.

## Results

### Overview

Forty-six interviews were conducted, representing perspectives from 25 clinicians across 8 CRHs. Most clinicians were geriatric psychiatrists (10/25, 40%) or geropsychologists (6/25, 24%). Other clinicians included neuropsychologists, clinical pharmacists, and a geriatrician (see Table 1). The CRH services primarily encompassed tele-GMH services, with later expansions including geriatric medicine. All models (n=23) included at least one GMH clinician and delivered synchronous telehealth services to home or clinic (see Table S1 in Multimedia Appendix 1). Teams provided services for veterans at spoke sites, including community-based outpatient clinics, smaller VHA medical centers, and video to home, including for patients receiving home-based primary care (HBPC) services. Two teams also provided telehealth services to community living centers (CLCs), VHA’s nursing home equivalent.

**Table 1.** Interviewee characteristics (n=25).

Characteristics	Interview phases		
	Time point 1 (n=23)	Time point 2 (n=13)	Time point 3 (n=11)
Discipline			
Geriatric psychiatrist	9 (39.1)	5 (38.5)	6 (54.5)
Geropsychologist	6 (26.1)	4 (30.8)	3 (27.3)
Neuropsychologist	4 (17.4)	1 (7.7)	0 (0)
Other clinician type^a^	4 (17.4)	3 (23.1)	2 (18.2)
Model type			
Consultative (2 regions)	8 (34.8)	3 (23.1)	4 (36.4)
Hybrid (4 regions)	10 (43.5)	7 (53.8)	5 (45.5)
Continuity (1 region)	2 (8.7)	2 (15.4)	2 (18.2)
Other model type^b^ (2 regions)	3 (13.0)	1 (7.7)	0 (0)
Interview duration (minutes), mean (SD)	39.35 (0.42)	38.06 (0.42)	47.35 (0.36)
Invitation response rate^c^^,d^, n/N (%)	23/24 (95.8)	12 (92.3)	11/12 (91.7)

a Other category was created to help keep data anonymized for professions with lower numbers (ie, clinical pharmacist practitioners, social workers, geriatricians).

b Other model types include gap coverage and tele-neuropsychology services.

c One interviewee completed Part 1 and Part 2 interviews at the same time point as they were transitioning to a different clinical resource hub (CRH) position; the interview duration is included in mean for Part 1 interviews.

d Another interviewee who was the founding clinician of tele-GMH delivered services prior to 2021 and only completed a Part 3 interview.

**Figure 1.**
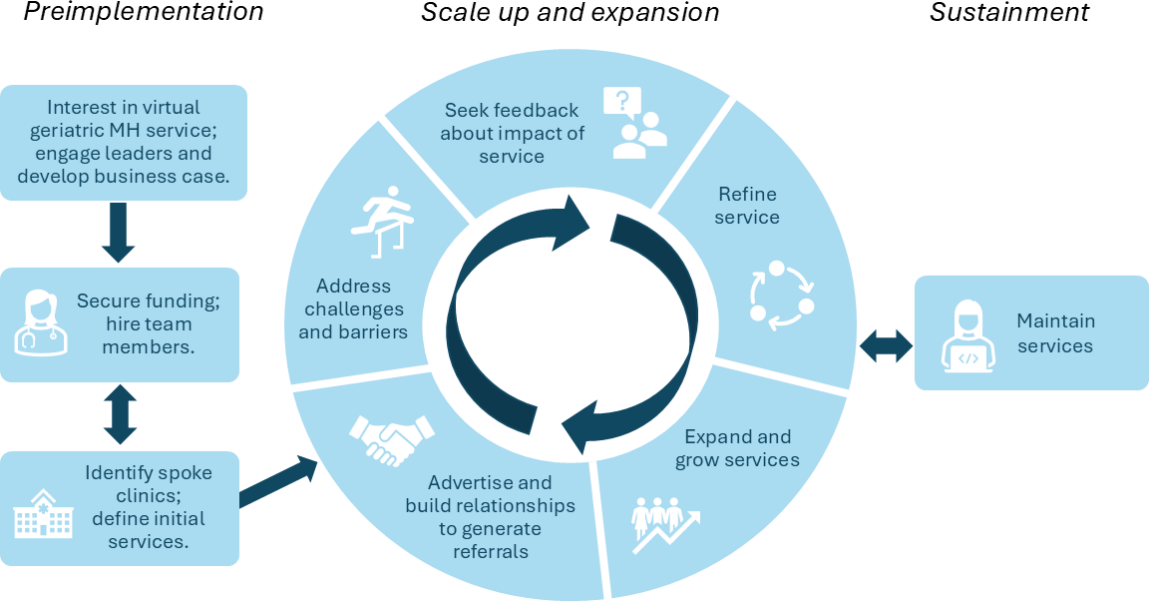
Overview of roadmap for implementation of tele-geriatric mental health services. MH: mental health

We identified the key steps taken to implement tele-GMH services, organized into three main phases: (1) preimplementation, (2) implementation scale-up and expansion, and (3) sustainment. As seen in Figure 1, the steps were cyclical with iterative changes over time. The following describes the steps and relevant determinants of implementation in each phase, with particular attention to the implementation of care for rural veterans.

### Preimplementation

#### Defining and Adapting the Innovation

The first tele-GMH service within the CRHs focused on a consultative model encompassing assessment, treatment recommendations, and limited follow-up appointments [20]. Variations on this approach emerged to include continuity-based services and a hybridized model of consultative and continuity services (termed “hybrid model”) [23]. During time point 1 interviews, we asked tele-GMH clinicians whether they had followed the originating consultative model in developing their services (guidebook available internally to VHA). Clinicians reported considering the model but making adaptations based on their own clinical experience in VHA settings to develop their service.

*…ultimately, we decided to do more of a shared intake and offer different modalities and then also provide some follow-up. We are staffed differently which allowed for the uniqueness of being able to provide follow-up.*

[ID 23, clinical pharmacist practitioner]

Others described developing their approach based on specific needs for services in their region, such as neuropsychology, continuity care for older veterans who needed care following the retirement of a local psychiatrist, or CLCs that needed psychiatry coverage. Thus, tele-GMH models can adapt to fit regional needs at start-up and to meet emergent needs over time (see Refining Service).

#### Building the Tele-GMH Team

Building the tele-GMH team required buy-in from and ongoing relationships with regional leadership in mental health and geriatrics to secure funding for clinical positions. In some regions, CRH leadership initiated building the tele-GMH teams, whereas in other regions, a self-identified champion (often a geriatrics or GMH clinician) advocated for establishing virtual services. Tele-GMH teams often attracted innovators with GMH expertise who were motivated to design new services. The first hires (ie, founding members of the teams) described how being involved in the design of the service gave them a sense of ownership and satisfaction in expanding access to GMH services for rural populations.

The CFIR characteristics of capability and motivation emerged as important constructs among clinicians hired into tele-GMH positions. Many described their prior VHA experience, including in settings such as HBPC, CLCs, and community-based outpatient clinics. Others who were hired from outside the VHA described their experience working in the community (eg, private practice and hospital) with older adults. Motivations included clinicians being drawn to serve the geriatric community and having the opportunity to use their training and work exclusively with older patients while addressing the national GMH clinician shortage. Many also acknowledged seeking out these positions to find a better work/life balance. One geropsychologist (ID 11) described their motivation for joining their CRH team:

*I've always enjoyed doing things with older adults that expand services for them, so I thought that this was a nice extension of that. Also, it was a completely virtual position, so I was hopeful that would give me more life balance and that kind of thing as well… I had been in a CLC setting prior and I was ready for a change after the whole COVID year and a half where nursing homes were really just hit so hard by that. So, all of those factors combined plus it just seemed like a good, exciting opportunity to do something new with geriatrics.*

[ID 11, a geropsychologist]

Others described the challenges of private practice, dealing with frequent cuts to Medicare/Medicaid reimbursement, and noting that it was “just getting harder and harder” with an influx of “corporate medicine” (ID 13, geriatric psychiatrist). Attracting clinicians into tele-GMH positions most often occurred through word of mouth from professional colleagues.

#### Specifying the Available Services and Identifying Initial Local Sites for Service Delivery

Prospective tele-GMH service offerings were decided at the outset by CRH leaders or the tele-GMH founding members through conversations with regional stakeholders about perceived needs and specific gaps in care pathways. Regional stakeholders included leaders in geriatrics and extended care, mental health, and primary care and their teams. Subject matter experts at sites were consulted, when possible, to avoid overlap with existing GMH services in a region. In some cases, needs assessments were conducted with the general mental health clinicians in the CRH, as they were the primary mental health clinicians for some rural clinics that experience substantial turnover of clinicians and thus would be the main referral sources for some teams. Table 2 summarizes the often-delivered services by the tele-GMH teams.

**Table 2.** Tele-geriatric mental health services. Other clinical services such as geriatrics evaluations for dementia, frailty, or multicomplexity may be delivered by geriatricians or advanced practice providers (APPs) along with other interdisciplinary team members.

Clinical functions/services	Clinicians who may deliver services^a^
Behavioral/environmental interventions to address disruptive or distress behaviors	Geropsychologist, geriatric psychiatrist, or geriatrician
Capacity evaluations^b^	Geriatrician, geriatric psychiatrist, neuropsychologist, or geropsychologist
Caregiver education related to psychiatric/neurocognitive disorders	Nurse, social worker, geropsychologist, geriatric psychiatrist, geriatrician, or advanced practice provider (APP) with geriatrics training
Cognitive assessment/neuropsychological assessment	Nurse, APP with geriatrics training, geriatrician, or geriatric psychiatrist could conduct brief cognitive assessments. Neuropsychologist or geropsychologist could conduct brief cognitive assessments or multidomain cognitive assessment batteries.
Diagnostic questions / second opinions related to neuropsychiatric presentations	Geriatric psychiatrist, geriatrician, APP with geriatrics training, neuropsychologist, or geropsychologist
Psychiatric medication recommendations and/or management	Geriatric psychiatrist, clinical pharmacist, geriatrician, or APP with geriatrics training
Psychotherapy specifically tailored for aging veterans	Geropsychologist, geriatric psychiatrist (may do brief counseling), or social worker
Psychotherapy for caregivers related to caregiver strain	Geropsychologist, geriatric psychiatrist (may do brief counseling), or social worker

a Clinicians from other disciplines, such as occupational therapists or speech and language pathologists, may also deliver these services.

b Not all teams provide this clinical service. It may depend on the specific question and state-specific rules and regulations.

A key facilitator to preimplementation was having a strong planning process, including establishing a sequence of steps and identifying key players at each local site. Telehealth coordinators played important roles in the startup as well to set up the necessary agreements for services. Delegating responsibility for each step and setting a timeline for the tasks streamlined implementation of services. Open lines of communication with other CRH clinicians who previously implemented services at a spoke site helped guide implementation. Barriers to implementation emerged when the specific processes, such as creating telehealth service agreements or identifying key points of contact, were not directly followed, which then led to delays in moving forward with service delivery.

### Implementation Scale-Up and Expansion

The scale-up and expansion of the tele-GMH services was an iterative process, as represented by the cyclical phase of Figure 1. See Table 3 for examples of the CFIR constructs related to the implementation process domain.

**Table 3.** Consolidated Framework for Implementation Research (CFIR) 2.0 implementation process domain analysis. One construct (assessing context) was not used when coding the data.

Implementation process domain construct	Summary of constructs within interview data	Illustrative quote
Teaming	Collaborating intentionally on tasks with referring clinicians (eg, attending staff meetings, discussing cases) helped develop rapport and established strong working relationships.	“[I attend] all of their [CLC]^a^ team meetings once a week, and discuss cases with them, active ones or referrals that are coming my way and that’s done for an hour every week” (ID 20, geriatric psychiatrist)
Assessing needs–innovation deliverers (ie, geriatric mental health [GMH] clinicians)	Assessing needs of innovation deliverers (GMH clinicians) guided expansion of services over time. Sometimes GMH clinicians required input from local clinicians to obtain information about local services.	GMH clinicians describe the barrier of being unfamiliar with local resources: “…we’re trying to refer them for adult daycare or, even a Senior Center or something like that. And we don’t know anything about what is there. I think that’s an ongoing challenge when not local and don’t know the local resources.” (ID 20, geriatric psychiatrist)
Assessing needs–innovation recipients (ie, veterans, caregivers, and referring clinicians)	Assessing needs of innovation recipients, such as patients with cognitive and/or sensory impairments, informed implementation and service delivery including time needed to troubleshoot technology and help patients connect to telehealth appointments.	“Many of these [patients] would just get canceled or no-showed… because we are aware, we take extra effort, I have none of my consults just get canceled because I can’t reach [them]. We have extra steps in place where we have someone on our team to go through the chart, just making sure we’re [calling] the right person and, maybe 75% of the time, turns out we’re not.” (ID 8, geriatrician)
Planning	Planning the steps in implementation was evident throughout the preimplementation and early implementation stages. This included planning the desired service to deliver, initiating services at a spoke site, or advertising services.	“There’s been some times where some sites seemed really eager for us to get started. And so, we’ll do things out of order, and then it just kind of like backfires because then we realized, oh, we don’t have all the safety contacts in place, or we didn’t finalize CVT availability is going to be …” (ID 4, geriatric psychiatrist)
Tailoring strategies	Tailoring strategies occurred regarding altering the planned menu of services offered to sites, adjusting time allotted to see patients, collaborating with other clinicians on shared patients, or adjusting other aspects of implementation.	“I am starting to do more briefer therapy now… while folks are inpatient. So that has been a huge change from the start of it.” (ID 7, geropsychologist)
Engaging–innovation deliverers (ie, CRH^b^ clinicians)	The opportunity to build an innovative service from the ground up attracted some CRH clinicians to their roles.	What drew some GMH clinicians to their role was: “the ability to kind of build something from the ground up, kind of like building a[n] airplane while flying it in a sense and being a part of something innovative.” (ID 14, social worker)
Engaging–innovation recipients (ie, veterans, caregivers, and referring clinicians)	Engaging referring clinicians and continuing to remind them of the service was a critical aspect of implementation. Advertising directly to veterans was found to be challenging due to the lack of a clear VA procedure on how to do this.	Continuing to remind referring clinicians of the service’s existence and purpose is key for awareness. “If the consult service rests on its laurels and doesn’t put their names out there all the time, we are private practice within the VA and they [referring clinicians] aren’t going to remember us or use us if they’re infrequent users of us and small outpatient CBOCs …so, we need to continue to let people know that we exist.” (ID 18, geriatric psychiatrist)
Doing	CRH clinicians adjust clinical processes and procedures throughout implementation to optimize efficiency and workflow.	Adding a phone line for patients improved coverage and responsiveness: “We’ve added is a phone line where patients can call in and leave a message and then our MSAs^c^ will give us the message and we can follow up… And then our nurse also created a dementia education packet that included a lot of the consistent resources that we were pulling for patients and information and educational tool.” (ID 23, clinical pharmacist practitioner)
Reflecting and evaluating–implementation	GMH clinicians reflected that note templates need to be standardized across sites for better workflow. GMH clinicians also described difficulties getting homebound or rural patients to attend in-person appointments like labs or EKGs.	Challenges came from trying to schedule lab work or an electrocardiogram for homebound or rural patients who are unable to come into the facility: “Lab work is difficult. They can go to lab and lab won’t come to them. And EKG [electrocardiogram], workups are difficult in [the] beginning if patient cannot go and nobody can come to them.” (ID 10, geriatric psychiatrist)
Reflecting and evaluating innovation	Telehealth services have been effective for older, rural patients and are valued by patients and caregivers alike. Referrals often come from clinicians who have referred to the service previously.	“[Caregivers] love [the support group] because hearing from other caregivers about how they’re surviving and that it’s normal to feel overwhelmed and all, that really helps.” (ID 15, geropsychologist)
Adapting	Adapting includes making modifications in response to changes that have been observed during implementation. Some sites decreased advertising efforts as the referral flow became steady and robust. Others added clinicians to the CRH GMH team or provided education.	“We did reach out to just a variety of primary care staff and it was very exhausting. It was like a press tour. It was a lot of extra work outside of work hours to do so much extra effort on top of patient care. But, I think it’s valuable because we’ve been on cruise control for a while, like we haven’t advertised for a long time, and we’ve had plenty of volume.” (ID 8, geriatrician)

a CLC: community living center.

b CRH: clinical resource hub.

c MSA: medical support assistant.

#### Advertising and Building Relationships

Generating referrals was crucial to the scale-up process. Referrals were initially increased through advertising and marketing to VA clinicians, and ongoing referrals were maintained through care collaboration with local teams. Outreach strategies included attending local and regional leadership meetings, attending local staff meetings, connecting individually with potential referring clinicians, and initiating regular contact with local sites (see Table S2 in Multimedia Appendix 1). Several tele-GMH clinicians used marketing materials, such as flyers, with service descriptions and other necessary information for sites to place consultation requests.

Despite the many facilitative strategies used to increase referrals, inconsistency in the naming of the tele-GMH service emerged as a potential barrier. Specifically, two key contributors were identified: (1) the slightly different naming conventions for consult orders across different instances of the VHA electronic health record (EHR) in each health care system, and (2) the uncertainty expressed by some interviewees when asked directly about the name of the service. This could have implications for advertising and maintaining a referral network.

#### Addressing Challenges and Barriers

The challenges that arose during implementation were grouped into 4 areas, including virtual care delivery, patient-related factors, availability of local services, and logistical challenges. Virtual care delivery challenges included needing to troubleshoot technology (eg, setting up assistive devices to be compatible with the technology, connectivity, and use of video platforms) before and during visits. Tele-GMH clinicians used different strategies to help facilitate access to and use of technology, including loaning VHA internet-enabled tablets (ie, Digital Divide Consult) and connecting patients with the VHA’s helpdesk to troubleshoot connecting to video visits. Telehealth clinical technicians or nurse case managers helped by conducting practice calls with patients and/or their caregivers as well.

Patient-related challenges emerged for specific populations, such as patients with cognitive impairment who do not have a caregiver present to assist with troubleshooting technology or to corroborate the patient’s medical history, or when working with patients with hearing impairment who are unable to reliably hear the clinicians through telehealth. Other complications arose when the point of contact listed in the electronic health record was not involved in the patient’s care. To overcome some of these challenges, tele-GMH clinicians collaborated with other VHA teams to obtain accurate information or coordinate visits. The CFIR construct of teaming, or intentional collaboration on tasks, included having local HBPC clinicians assist patients in logging on to a tablet or phone for the tele-GMH appointment.

Additional challenges arose regarding the availability of local resources. Some rural veterans had limited access to local primary care or specialty care. This made it challenging for those veterans to obtain timely and consistent medical care, which also impacts their mental health care. One geropsychologist (ID 15) described difficulties in trying to get in touch with local clinics.

*It’s scary to me, the risks that are inherent in that [scarce resources in rural areas] and feeling so separate from it and limited in what I can do, you know, because I refer people to the patient advocate. I provide those contact numbers. I really encourage them to speak up for themselves and then they'll tell me like, ‘I've called [local VHA clinic] 12 times and no one’s called me back.’ And you know, you just feel like your hands are tied and that’s really hard for my morale and their morale.*

[ID 15, a geropsychologist]

Potential solutions included working with local social workers or with the CRH nurse case managers to identify local resources.

The last challenge centered on logistics, clinical processes, and procedures. Tele-GMH clinicians described making small adjustments after they began providing services. These iterative changes, captured by the CFIR construct of doing, led to changes in patient-clinician communication (eg, adding a phone line where patients could leave messages for a medical support assistant who triages requests), workload balance among team members (eg, adjusting intake appointments), improving within-team communication (eg, developing a format for internal huddles and patient discussions), and communication with sites (eg, developing internal communication channels with each local site).

#### Seeking Feedback About Impact of Service

Tele-GMH clinicians described ways in which they receive feedback about the impact of their services, captured by the CFIR construct of reflecting and evaluating. Positive feedback about the service encompassed effects on veterans (acceptability of telehealth, convenience of telehealth, and positive impact on quality of life); caregivers (involvement in care and their appreciation of services); referring clinicians (assistance with patients with complex needs); and access to specialty care (see Table 4).

**Table 4.** Tele-geriatric mental health service impacts.

Theme	Definition	Illustrative quote
Increased acceptability of telehealth for older veterans	Older veterans had positive responses to telehealth technology and preferred telehealth. Referring clinicians expressed initial skepticism about telehealth being effective; however, this was overcome as the virtual team proved to be helpful.	“There’s still this culture or stigma around the use of technology and older adults…I think we’re seeing that, yes, there’s barriers and challenges for sure. It’s a unique population with unique needs, but we absolutely can make this work.” (ID 6, geropsychologist)
Telehealth is convenient for older veterans	The telehealth service enables patients with age-related health conditions to avoid driving far distances by offering video-to-home appointments (VA Video Connect [VVC]) and video appointments to local clinics (Clinical Video Telehealth [CVT]).	“…saving them having to drive to places that are further away, whether they’re doing VVC visits from their home, which obviously is the easiest way to do it… it’s been a lot easier for them not to have to drive as far as they might otherwise.” (ID 20, geriatric psychiatrist)
Caregivers are often involved in and value the services	Patients are the focus of the service; and many have stressed and overwhelmed caregivers. Caregivers benefit from direct services such as caregiver groups or brief therapy, but also from education from clinicians during the veterans’ appointments.	“[Caregivers] don’t feel like they can be honest about how scary [caregiving] is and how taxing and overwhelming it is because they don’t want to burden other people. And they feel a sense of shame sometimes because they feel like they’re supposed to just be up for it all and never complain.” (ID 15, geropsychologist)
Positive effects on quality of life for veterans, particularly those with cognitive impairment	The service has resulted in patients getting high quality care managing dementia, behaviors and neuropsychiatric symptoms. This care extends to quality of life for the veterans.	“…with dementia there’s no cure, but we can give them the best quality of life possible and the best time with their loved ones and caregivers, the best time with their family member with dementia that we can and, and that’s the ultimate goal for us.” (ID 23, clinical pharmacist practitioner)
Referring clinicians appreciate the services for patients with complex needs	This service benefits referring clinicians by reinforcing their decisions and providing additional guidance when needed. Education to direct care staff also helps them manage veterans with disruptive or challenging behaviors and conflicts with staff and other patients.	“…anticipating questions that they might not realize they might have. And I think also just trying to alleviate concerns or kind of anxieties about, you know, in particular with dementia-related behaviors to manage, just reinforcing that I’m there to provide help when needed. So, not having to worry alone.” (ID 4, geriatric psychiatrist)
Services increase access to needed expertise	These services increased access to geriatrics care for highly rural patients in under-resourced areas who may otherwise “fall through the cracks” in health care systems.	“[Local clinicians] may want to refer that Veteran on to a higher level of care or to community care, and that usually delays care a lot versus having that geriatric mental health specialist or geropsychologist available. You can see that patient right away. So again, it’s just really makes care a lot more accessible and efficient.” (ID 1, geropsychologist)

Constructive feedback was elicited when tele-GMH clinicians connected with referring clinicians to discuss the recommendations given. During those conversations, tele-GMH clinicians learned more about situations in which the referring clinicians did not follow the recommendations. One geriatric psychiatrist (ID 20) described learning that the consultative care approach conflicted with referring clinicians’ preferences.

*I've gotten some feedback from the providers or at least in the consultation that they were really hoping for somebody to kind of take over the prescribing and be the provider for the patients going forward. I just had a consult right before this call up in [STATE] where it’s a primary care doctor who is not at all comfortable prescribing the meds and was really looking for me to kind of take over that… my role hasn't changed, but maybe, we should rethink that and see what’s best for it. That’s probably the biggest thing.*

[ID 20, a geriatric psychiatrist]

The tele-GMH team reflected on the impact of their service model on patients. For example, one clinician using a consultative care approach noted that this model allows patients to be seen within 1-2 days compared with 1-2 months. Clinicians using a hybrid model focused on stabilization or more continuity services described the challenges with longer wait times (4‐8 wk). This latter observation led to tailoring strategies of increasing the number of intake slots and broader adaptations such as advocating to hire more clinicians to the team.

#### Refining Service

Refining the services largely consists of the CFIR constructs of tailoring and adapting the service delivery in response to feedback, observations, and emergent needs. One such change was to tailor the specific services offered according to the local needs. For example, a geropsychologist who initially intended to provide behavior plans and assessments expanded their services to include brief psychotherapy (4‐5 sessions). Tele-GMH clinicians described setting aside time to intentionally collaborate on care with local clinicians through teaming strategies such as attending weekly behavioral huddle meetings in CLCs. Other adjustments focused on how the team divided up cases from the referring sites. For example, one service consisting of multiple geriatric psychiatrists learned that assigning each psychiatrist a CLC to cover was less confusing and prevented mixing of treatment styles and medication choices. Teams also described that refinement encompassed learning what expertise each discipline may bring to the team, which helped their team work more effectively together. One site found that their processes became more efficient when the newly hired nurse case manager began conducting prescreening questions prior to the patient’s initial appointment.

#### Growth and Expansion

Growth and expansion of services encompassed adding spoke sites to be served by tele-GMH teams, offering new services (eg, caregiver groups), and hiring more clinicians. One team developed a virtual interprofessional cognitive assessment clinic in response to an emergent need within a health care system following their loss of a dementia assessment clinic after the lead clinician’s retirement. For teams experiencing increasing referrals or additional sites, they met these demands by hiring additional clinicians, such as psychologists, geriatric psychiatrists, clinical pharmacists, and nurse case managers. However, moving toward interprofessional care led to logistical challenges using different disciplines (eg, how neuropsychology fits into the service, how to efficiently use a new nurse manager). A geriatric psychiatrist (ID 16) described challenges in equitably assigning workload and corresponding with referring clinicians.

*We had to do some work because you've got three different providers covering five states, multiple different sites. And if we're all seeing one or two people here or there per week, it gets challenging for the primary providers there to know ‘Who just saw my patient?’ and ‘Who do I reach out to?’*

[ID 16, a geriatric psychiatrist]

Notably, not all teams focused on expansion. Some described stability in their workload, which led to the team decreasing advertising due to a steady stream of consultations. Others reported identifying systems-based challenges, namely serving homebound or rural patients. One geriatric psychiatrist (ID 10) described the need for a social worker to help with identifying local aging services.

*I'm a single man army here. I don't have any social worker… I know there’s [a] psychologist, but sometimes getting information about resources locally [is hard]. Again, it’s not one clinic, [it’s] multiple clinic[s], I go to so many, so I don't know what exactly is there.*

[ID 10, a geriatric psychiatrist]

A geropsychologist (ID 15) noted the absence of geriatric medicine services as a challenge:

*…if you’re going to integrate geriatric mental health, and neuropsychology, you need the primary care in place. It’s kind of crazy to be siloed as geriatric mental health when there’s no geriatric physical health services in the [facility].*

[ID 15, a geropsychologist]

### Maintenance and Sustainment of Service

The maintenance and sustainment phase encompassed stability in the interprofessional teams and focused on collaboration with local clinicians. Other key aspects of the maintenance phase were reflections on the impact of the service on patients, caregivers, referring clinicians, and an overall increased access to geriatrics specialty care, as displayed in Table 4, and found within the CFIR construct of reflecting and evaluating.

Key facilitators to maintenance of services were positive interprofessional relationships, including mentoring new team members. One geropsychologist (ID 7) said of the founding tele-GMH team member:

*I think that she has been a gift to me with regard to just her assistance in taking me under her wing and bringing me along with her in an already established little program that she had.*

[ID 7, a geropsychologist]

Similarly, a clinical pharmacy practitioner (ID 12) felt their team nurse was “fantastic… she’s been instrumental for some success” because the nurse holds multiple roles, like helping psychology with overflow work, triaging patients, and contacting veterans and facilitating connecting them with tech help. Hiring of support staff, such as a dedicated medical support assistant to assist with scheduling, was deemed to be very supportive, enabling the teams’ ongoing success.

## Discussion

### Principal Findings

Using qualitative methods with multiple interviews conducted with tele-GMH clinicians across a 3-year period, we identified the key activities and determinants of success across three phases: (1) preimplementation, (2) implementation scale-up and expansion, and (3) sustainment of tele-GMH services for older veterans.

The preimplementation phase encompassed the crucial steps of engaging leaders, securing funding/hiring, and defining services. A key determinant of success during this phase was the use of adaptations to the tele-GMH model to meet local needs and priorities. Also important were the characteristics of the individuals involved in developing and delivering the tele-GMH services. The CFIR domains of capability (ie, expertise in GMH) and motivation to care for rural and underserved older veterans were factors that led clinicians to these CRH positions. Expanding virtual positions may be one way to address the persistent geriatrics workforce shortage and enable specialist clinicians to serve the population they trained to serve. Moreover, remote work has been found to be associated with low burnout, particularly for female health care workers [34]. Thus, expanding virtual positions may help individual clinicians while also filling the geographic need for geriatrics care in rural regions [35].

The implementation scale-up and expansion phase was cyclical and iterative, focused on building a referral network and expanding or maintaining services offered to older veterans. Key determinants in this phase included building and maintaining relationships with local clinicians, teaming (or intentional collaboration), doing (making iterative changes to processes), tailoring services for sites, and adapting services in response to feedback. This fits with findings on the importance of partnership alignment, communication, and feedback as determinants of implementing comprehensive geriatrics assessment [36]. A key determinant to both preimplementation and implementation was having the tele-GMH team embedded within CRH, which allowed the clinicians to take advantage of CRH operational knowledge to initiate services. This likely allowed the tele-GMH clinicians to focus on building relationships and designing their program while CRH staff guided them in the logistics of telehealth service delivery. This echoes previous work that identified the critical role of administrative staff in supporting virtual services [37].

Tele-GMH clinicians described periods of maintenance followed by refinement and growth in the third phase. Barriers to implementation, including patient-level factors, virtual care obstacles, and unfamiliarity with local aging resources, were addressed using refinement to processes and engaging nurse case managers, social workers, and telehealth clinical technicians. As found in our previous work with surveys of referring clinicians [22], several barriers impede the uptake of services, including lack of clarity about consultation process logistics (ie, who can place consultations and from which settings), perceptions that older veterans may not benefit from telemedicine, and not following tele-GMH clinician recommendations. Facilitators of the maintenance phase include clear messaging, teaming, relationship building within teams, and care coordination. The maintenance phase warrants future study to better understand the mechanisms underlying high-functioning remote health care teams and how these teams communicate with local VHA and community clinicians. This communication and care coordination is critical as many rural veterans use both VHA and community care [38]. Furthermore, there is a need to understand patient perspectives as they pertain to working with remote teams. A mixed methods study found unclear and overlapping health care clinician roles and limited care coordination to be barriers to the effective virtual primary care for patients with chronic diseases [39].

The growing shortage of both mental health and geriatric clinicians demands innovative and scalable solutions and programs to address the needs of older adults. This work dovetails with calls for integrated geriatric clinical service lines rather than disease-focused service lines [35]. However, due to workforce shortages [2,3] and the limited availability of geriatrics clinicians in rural areas, virtual care models must be considered. Tele-GMH has the potential to be such a program due to the adaptability of the model, sustainability of service delivery with embedded administrative support, and attraction of specialist clinicians to serve older veterans. Strengths of our study include using CFIR to identify determinants of success and barriers to implementation that can be applied in similar program development in other health care systems. Although the tele-GMH service may be adapted to care for veterans with complex needs, broader initiatives to transform health care, such as “Age Friendly Health Systems,” must be pursued to improve care for all older adults [40].

### Limitations

Several limitations to this qualitative evaluation warrant consideration. First, the findings are limited to perspectives on VHA and thus may not directly apply to other health care systems. Second, we do not include quantitative outcomes or perspectives from patients or caregivers, which limits the interpretation of the acceptability of the findings and impact on service recipients. Third, our findings rely on tele-GMH clinician self-report, which may not fully capture all VHA perspectives in this report (ie, referring clinician, VHA leadership), although those perspectives have been reported elsewhere [21-23]. Fourth, we did not interview every tele-GMH clinician at each time point, potentially biasing our findings. Fifth, our findings may be biased by sampling of clinicians who chose to work in these roles in the CRH. Sixth, the rapid analytic approach fit with our team-based synthesis; however, it does not allow for the line-by-line analytic depth of traditional qualitative coding approaches.

### Conclusions

Our findings provide evidence of implementation factors related to virtual GMH care, which was noted as a main weakness in the broader literature on geriatrics care models [41]. Similar processes emerged across regions in the development and sustainment of tele-GMH services, which allowed for the creation of a unified roadmap. The roadmap provides a pathway that other regions may follow to create tele-GMH services to enhance access to specialty care for aging rural veterans. Our findings underscore the role of VHA as a learning health system [42] where experts and innovators can pilot and refine new models of care.

## Supplementary material

10.2196/95286 Multimedia Appendix 1 Supplemental materials contain researcher reflexivity statement and supplemental tables 1 and 2.

## Abbreviations

CFIR: Consolidated Framework for Implementation Research
CLC: community living center
CRH: clinical resource hub
GMH: geriatric mental health
HBPC: home-based primary care
VHA: Veterans Health Administration
