# Supplementary material for: A Roadmap for Implementing Virtual Geriatric Mental Health Services for Rural Veterans: Qualitative Longitudinal Evaluation
Source: JMIR Aging. 2026 Jun 16;9:e95286. doi: 10.2196/95286 (PMC13271587; doi:10.2196/95286)
Supplement: Multimedia Appendix 1 [file aging-v9-e95286-s001.docx]

**Supplemental Materials**

**Researcher Reflexivity Statement**

The evaluation team was known to the clinicians delivering geriatric mental health services as the evaluation team facilitated a periodic community of practice meeting as part of their funded pilot project from the Office of Rural Health. This familiarity with the team may have led them to being more likely to agree to participate in the interviews and share information with our team. Each research team member summarized their individual characteristics and experiences that may influence their reflexivity below.

Christine Gould is a board-certified geropsychologist at the VA Palo Alto GRECC. She identifies as a white female. She trained in geropsychology at West Virginia University where she obtained her MS and PhD and has worked at the VA Palo Alto for 15 years. Her evaluation work is informed by her research and clinical interest in using technology with older adults, her interest in increasing mental health access for older adults, and her interest and experience in the Veterans Health Administration.

Marika Blair Humber is a psychologist with a PhD in clinical psychology from Palo Alto University. She identifies as female and Asian American. After completing internship at Gulf Coast Veterans Health Care System – Biloxi and a caregiver-focused health services research fellowship at VA Palo Alto Health Care System and the Elizabeth Dole Center, she transitioned to program evaluation at VA in 2022. She has served as project manager and qualitative analyst on several VA projects. Her evaluation work is informed by her background in clinical psychology and research on informal caregivers navigating healthcare systems.

Lynsay Paiko, a white woman, is a licensed psychologist and Advanced Fellow in Geriatrics at the VA Palo Alto. She completed her pre-doctoral internship focused on geropsychology and clinical geropsychology postdoctoral fellowship at the VA Palo Alto. Her clinical training complements her evaluation interests and work related to increasing mental health care access for older adults.

Chalise Carlson identifies as a white female, with a professional background in research and experimental psychology, as well as geriatrics operations. She has been working at the VA for the past seven years. Here training and experience influence my perspective and approach to both research and operational decisions within the VA context.

Marisa-Francesca Lindsey identifies as a mixed-race Filipino and White female with a professional background in clinic operations and coordination. She holds a Bachelor of Science degree in Exercise Science, has over 13 years of experience within the VA, and currently works as Program Coordinator for program evaluations. Her education, professional training, and long-term involvement in the VA influence the perspectives she brings to research and the way she interpret information and interactions within the organization.

Althea Lloyd, is a clinical geropsychologist with the VISN 7 Clinical Resource Hub.  She has worked with the Veteran’s Health Administration for 18 years, in multiple settings of care. She identifies as a black female of Caribbean descent. Her professional training and clinical experiences have focused primarily on improving care of older adults and the intersectionality of cultural factors.

Ranak Trivedi, is a health psychologist and health services researcher with expertise in caregiving and dyadic self-management. She was raised in India and educated at Duke University and uses a culturally attuned, family-centered approach to her work. She holds appointments as an Associate Professor at Stanford University School of Medicine and as an investigator at the Center for implementation to Innovation at the VA Palo Alto Health Care System. She also has lived experience as a caregiver.

Amanda Peeples, a white woman, holds a PhD in gerontology from the University of Maryland, Baltimore County. She has worked in VA health services research and evaluation for over ten years on projects spanning mental health, chronic disease, recovery, and age-friendly health care. She is currently a core investigator and qualitative methodologist at the Center for Healthcare Evaluation, Research, and Promotion at the Corporal Michael J. Crescenz VA Medical Center in Philadelphia, PA.

**Table S1.** Overview of Tele-Geriatric Mental Health Models and Service Delivery

| **Model** | **Approach to care** | **Discipline(s) on Team** | **Service modality** | **Patient Care Settings** |
| --- | --- | --- | --- | --- |
| Consultation with time limited follow-up | Consultative service with limited follow-up. Consultation may be for medication recommendations, diagnostic clarification, cognitive assessments or capacity assessments from geropsychologist or neuropsychologist, time-limited psychotherapy with geropsychologist, and behavior plans. | - Geriatric Psychiatrist - Geropsychologist - Neuropsychologist - Clinical Pharmacist - Nurse Case Manager | - Synchronous telehealth (video to home, video to clinic, phone) - e-consult | Outpatient, HBPC, CLC, inpatient (e-consult only) |
| Hybrid (consultation and continuity services) | Both consultation and continuity care services are provided. The disciplines on each team, settings served, and specific services delivered vary by hub. Teams range from 1 to 5 providers in size. For some teams, provision of consultative versus continuity care depends on the local clinic being served. Another variation is provision of episodes of care focused on stabilization of geriatric mental health concerns prior to return of care to referring provider and/or primary care. | - Geriatric Psychiatrist - Geriatrician or Advance practice nurse - Geropsychologist - Neuropsychologist - Social Worker - Clinical Pharmacist - Nurse Case Manager | - Synchronous telehealth (video to home, video to clinic, phone) - e-consult | Outpatient, HBPC, CLC |
| Continuity | Continuity care services are provided consistent with typical panel management approach focused on following the patient over time. Services include medication management and psychotherapy. Hub also has a robust tele-neuropsychology^1^ service separate from geriatric mental health services. | - Geriatric Psychiatrist - Geropsychologist | - Synchronous telehealth (video to home, video to clinic, phone) | Outpatient |
| Gap Coverage^2^ | Continuity services and consultation delivered where gaps in care were identified in VHA region. | - Geropsychologist | - Synchronous telehealth (video to home, video to clinic, phone) | Outpatient/HBPC |

Notes. CLC = Community Living Centers, HBPC = Home-based primary care, VHA = Veterans Health Administration. ^1^Tele-neuropsychology services that were not integrated with geriatric mental health teams are not described herein as a separate geriatric mental health model. ^2^Gap coverage model was used during FY2023, but not used at present by the geriatric mental health teams in CRH.

**Table S2.** Different Outreach Strategies Utilized by Tele-Geriatric Mental Health Teams

| **Forms of Outreach** | **Description** | **When/How Often?** | **Exemplary Quote(s)** |
| --- | --- | --- | --- |
| Chatting with providers via Teams | Describing service to new referral sources or checking in with providers who have referred previously to connect, ask questions, offer help, and see if there are any new cases to refer. | At initial outreach/advertising step or after completing a referral for a provider, follow up as needed according to stream of consults/workload | “We do teams, we let the person know via Teams message or through cosign note, but primarily Teams message, ‘hey, I finished that consult for you. If you have questions, please let me know.’ Or if it's a, particularly if it's a follow up and some are follow-ups, you know, if it's a pretty complicated patient, I will say ‘hey, do you have time to touch base with me? I just saw a patient of yours.’” (ID 18, geriatric psychiatrist) |
| Maintaining regular contact with sites | Checking in with the primary point of contact at the site where referrals are generated. | After initial contact meeting, check in periodically and as needed according to stream of consults/workload or attend clinical huddles | “First is figuring out like who's who and, who are the main contacts at the respective sites that you should maintain contact with. For me, early on it was a matter of asking, what are their recurring like team meetings that I could like join, and maintaining a presence there, has really been vital to integrating myself into the team, in that respect. And for the CLC, in particular, that has worked well. And we've made some modifications again from site to site.” (ID 4, geriatric psychiatrist) |
| Meeting with providers in-person | Traveling to visit sites if possible. Visiting CLCs to meet with staff may be particularly beneficial. Offering assistance and asking if there are new eligible patients to refer. | Infrequently, possibly once a year. When possible, visit a site long enough to meet with staff working different shifts. | “Traveling to the different sites, [CRH geriatric psychiatrist] was very good about doing that pre-COVID. Just again, it's different than getting your name out there. Actually, in house, you're talking with people you're meeting with people and so that's something also on our plan to continue to bolster services.” (ID 7, geropsychologist |
| Sending emails | Use combined with other strategies; insufficient alone. | Use with initial meetings and formal follow up contacts. | “So we did a huge like, branding [CRH team lead=] called it a press tour, so we just, like, sent all the local sites a bunch of emails. And then I got in contact with all like the neuropsych folks. And then just kind of built relationships with them, telling them like who we are, what we're here to do, that we're really just trying to be like an additional like safety net for them.” (ID 19 neuropsychologist) |
| Sharing success stories and milestones | Gather testimonials or success stories, share a “good news story”, and share milestones with referring providers. | As opportunities arise (e.g., call for articles in newsletters), when milestones are reached, or when cases are treated successfully | “When we hit our 100th consult, we sent out a thank you so much for allowing us the opportunity to help serve your veterans to the sites we were at and… sharing good news stories in a way to educate them of who we will take or how we've worked with patients to help them kind of understand the uniqueness of our program.” ((ID 23, clinical pharmacist practitioner) |
| Using marketing materials | Disseminate as part of other strategies. Contains service descriptions and other necessary information. | Use with initial meetings and formal follow up contacts. Use alongside *sending emails* and *creative advertising* | “…sending emails to individuals with a couple of sheets [attachments]. One sheet was more basic of what I did. The other sheet, the other document, was a bit more in detail of what I did, just informing folks about the services that I'm able to provide.” (ID 7, geropsychologist) |
| Virtually attending staff and/or leadership meetings | Attending staff and/or leadership meetings and huddles to introduce the providers from the CRH team, describe services and answer questions (5-15 min). Sharing marketing materials via Microsoft Teams or email. | When initiating services at a site, then periodically to remind staff of available services, share any changes to services, etc. (see *initiating regular contact with sites*) | “We have gotten in on a lot of their Teams meetings just to kind of explain what our services are and how to, you know, access the consult menu in the chart and put the consult order in, all of that” (ID 20, geriatric psychiatrist) |
